# Cyclical Electrical Field‐Flow Fractionation for Retention Characterization of Per‐ and Polyfluoroalkyl Substances

**DOI:** 10.1002/ansa.70115

**Published:** 2026-10-24

**Authors:** Fatemeh Momeni, Jenika Mifflin, Himanshu Sant, Bruce K. Gale

**Affiliations:** ^1^ Department of Mechanical Engineering University of Utah Salt Lake City, Utah USA; ^2^ Department of Environmental Engineering University of Utah Salt Lake City, Utah USA

**Keywords:** conductivity detection, electrophoretic mobility, environmental contaminants, perfluoroalkyl sulfonates, retention ratio

## Abstract

Per‐ and polyfluoroalkyl substances (PFAS) are environmentally persistent contaminants that are difficult to monitor and separate because of their chemical stability and diverse physicochemical properties. In this study, the retention behavior of several PFAS, including perfluorooctane sulfonate, perfluorohexane sulfonate (PFHxS‐K), perfluorobutanesulfonate (PFBS), hexafluoropropylene oxide dimer acid, and perfluorononanoic acid, was investigated using Cyclical Electrical Field‐Flow Fractionation (CyElFFF). The selected compounds represent PFAS with different chain lengths, functional groups, and environmental relevance. Field‐off injections established the void response, while application of a cyclical electric field produced analyte‐dependent retained peaks, demonstrating that CyElFFF can induce measurable retention for multiple PFAS compounds. Voltage and frequency sweeps showed that retention behavior is sensitive to the applied electrical conditions, with increasing voltage generally increasing retention and frequency changes producing smaller but measurable changes in peak position and shape. PFBS concentration studies showed that increasing concentration primarily increased peak height, while retention ratios and peak positions changed only slightly over the tested range. The PFBS/PFHxS‐K mixture was not baseline‐resolved under the tested conditions. Because the mixture and individual standards were not matched for total PFAS concentration, conductivity, or ionic strength, the cause of the shifted mixed‐sample response could not be determined. In addition, conductivity detection could not specifically identify the contribution of each compound to the mixed profile. Overall, these results show that CyElFFF can induce and modulate retention for multiple PFAS, while baseline mixture separation will require further optimization of operating conditions and reduction of non‐ideal channel interactions. Experiments were carried out at part‐per‐million concentrations to establish fundamental transport behavior rather than to demonstrate trace‐level detection.

## 1 Introduction

The persistence of per‐ and polyfluoroalkyl substances (PFAS) in the environment and their possible negative effects on human health are well documented [1, 2]. PFAS build up in biological and ecological systems and are resistant to degradation due to their strong carbon‐fluorine bonds. PFAS are found in many settings and have uses in both consumer and commercial products [3]. There are thousands of different PFAS used in diverse industries with a range of structures and potential toxic interactions. The majority of available PFAS toxicity data concentrates on a small number of legacy chemicals, including perfluorooctanoic acid (PFOA) and perfluorooctane sulfonate (PFOS) [4]. Experimental animal research and epidemiological studies confirm associations between exposure to these substances and a range of health problems, including cancer, liver disease, and thyroid dysfunction [5]. These environmental and health concerns, together with the structural diversity of PFAS, motivate the development of analytical methods for PFAS characterization and separation.

Field‐flow fractionation (FFF) offers one potential platform for such characterization and separation. FFF involves the application of an external field perpendicular to a thin, wide channel, which drives analytes toward one channel wall. As a result, analytes experience different flow velocities depending on their interaction with the field and the channel velocity gradient, enabling differential retention and potential separation. FFF uses a single‐phase carrier solution, which lowers the possibility of sample alteration compared to traditional two‐phase methods. Particles can be sorted according to size or electrophoretic mobility using FFF variants such as Electrical FFF (ElFFF), which applies an electric field to achieve a separation based on electrophoretic mobility [6, 7, 8, 9, 10]. Given the difficulties presented by the chemical nature of PFAS, we hypothesize that ElFFF could be an effective tool for analysis and collection and may enable methods to study the use of electric fields in characterizing different PFAS subtypes.

Cyclical ElFFF (CyElFFF) is a variant of ElFFF that employs an alternating electric field (alternating current [AC]) rather than a constant direct current (DC) field. This modification was introduced to overcome the limitations of low effective field strength observed in conventional ElFFF systems. Reversing polarity each half‐cycle limits electrode polarization and maintains a stable effective field. By contrast, a steady DC field decays as the electrode double layer charges, weakening the driving force. Cyclic excitation (including square waves) thus provides more consistent field strength for ElFFF operation [11]. In CyElFFF, particles migrate cyclically between the channel walls instead of accumulating at a single wall, which can improve separation performance and reduce polarization effects. The retention behavior of particles is governed by their electrophoretic mobility as well as by the amplitude and frequency of the applied field [12]. However, there is a dearth of literature on PFAS separation and retention in CyElFFF with respect to critical parameters such as sample concentration, pH, injection volume, and flow rate, as well as electric‐field properties like voltage and frequency.

We characterize and quantify single‐compound PFAS retention in CyElFFF (PFOS, perfluorohexane sulfonate [PFHxS‐K], perfluorobutanesulfonate [PFBS], GenX, and perfluorononanoic acid [PFNA]) by injecting standards under a fixed carrier, pH, flow rate, and injection volume. For each analyte, we measure retention time, compute the retention ratio (relative to the void time) and separation resolution, then run one‐factor sweeps of applied voltage, field frequency, and sample concentration while holding other conditions constant. This protocol identifies operating conditions that affect single‐standard retention and documents the lack of baseline resolution for the PFBS/PFHxS‐K mixture under the tested conditions without assigning a mechanism to the altered mixture profile. Together, these results characterize the fundamental retention behavior of individual PFAS under cyclical electric fields and identify the operating windows and non‐ideal effects that currently limit mixture separation. All experiments were performed at part‐per‐million concentrations to probe transport behavior, so this work is a proof of concept for field‐induced PFAS retention rather than a trace‐level detection method, given that environmental regulatory thresholds for PFAS lie in the part‐per‐trillion range.

## 2 Theory

CyElFFF is a technique where an AC dynamic electric field manipulates charged particles. It extends the capabilities of traditional ELFFF by utilizing alternating electric fields to counteract polarization effects that are typically present in DC techniques. The alternating electric field directly influences particle movement based on its electrophoretic mobility (*μ*), thereby determining particle trajectory in the flow channel and hence separation and retention. Particles of different mobilities from the electrophoresis track have different paths, which results in them being retained and potentially separated effectively when they leave the system at distinct time points.

The experimental retention ratio, (*R*), is defined as the ratio of the extra‐column‐corrected void time of a non‐retained species to the extra‐column‐corrected retention time of a retained species:

(1) $$R=\frac{t_{0,obs\;}-t_{d}\;}{t_{R,obs}-t_{d}}$$

where $t_{0,obs\;}$ is the detector‐observed void time, $t_{R,obs}$ is the detector‐observed retention time, and $t_{d}$​ is the extra‐column transit time. Retention time was consistently defined as the time at the peak apex.

Because *R* is measured directly from chromatograms, it is a key metric for interpreting transport and separation mechanisms in FFF and related chromatography methods [11].

*R* varies depending on the mode of movement as determined by the parameters of the applied electric field, which can be characterized by the dimensionless retention parameter $\lambda_{0}$, a measure of particle motion; when $\lambda_{0}$ is less than one, the system is in Mode I and particles do not cross the channel fully; when $\lambda_{0}$ is greater than one (Mode III), the particles reach the opposite wall and spend some time there before reversing direction; when $\lambda_{0}$ is equal to one (Mode II), the particle reverses direction upon arrival at the wall. $\lambda_{0}$ can be calculated by:

(2) $$\lambda_{0}=\frac{\mu V_{B}}{2fw^{2}}$$

where *f* is the frequency of the applied electric field, *μ* is the electrophoretic mobility of the particle, *w* is the channel spacer thickness, and *V_B_* is the effective voltage across the bulk phase. Quantitative assignment of the CyElFFF operating mode requires independent determination of the analyte electrophoretic mobility and the effective electric field in the bulk of the channel. In the present study, the electrophoretic mobility of the PFAS analytes under the experimental carrier conditions and the effective bulk‐channel voltage were not independently measured. Therefore, $\lambda_{0}$ was not calculated, and Mode III operation was not established quantitatively. The experimentally measured retention ratios describe the extent of retention but were not used alone to assign the operating mode. Comparisons with Mode III theory are consequently qualitative.

In mode I, *R* is given by:

(3) $$R=3\lambda_{0}\left(1-\frac{2}{3}\lambda_{0}\right),\;\left(\;\lambda_{0}\leq 1\right).$$

In mode III $\;(\lambda_{0}\geq 1)$:

(4) $$R=\frac{1}{\lambda_{0}}$$

These two expressions join at $\lambda_{0}$ *=* 1 (Mode II), yielding a continuous function. In this framework, the only particle property governing retention is the electrophoretic mobility. This single‐mobility description is an idealization. In practice, analyte‐wall interactions, finite double‐layer relaxation, and possible analyte aggregation can produce behavior that departs from this model, including more than one retained peak for a nominally single analyte. We return to these non‐idealities when interpreting the experimental fractograms.

To estimate the magnitude of the effective electric field within the CyElFFF channel, Gale proposed an equivalent circuit model that accounts for both the bulk solution and electrode interface effects [11, 13]. In this model, the field strength is influenced by the applied voltage, frequency, and the impedance contributions from both the bulk electrolyte and the double‐layer polarization at the electrodes [14]. The effective electric field can be calculated using:

(5) $$E_{\mathrm{eff}}=\frac{VR_{B}}{w}\sqrt{\frac{1+{\left(2\pi fR_{DL}C_{DL}\right)}^{2}}{{\left(R_{B}+R_{S}+R_{DL}\right)}^{2}+{\left[2\pi f\left(R_{B}+R_{S}\right)R_{DL}C_{DL}\right]}^{2}}}$$

as described in [15], where *V* is the applied voltage across the electrodes, *f* is the frequency of the cyclical voltage waveform, *R_S_* is the resistance of the voltage source, and *w* is the height of the separation channel. *R_B_* denotes the bulk resistance of the electrolyte, while $R_{DL}$ and $C_{DL}$ represent the resistance and capacitance of the electrode polarization (double‐layer) region, respectively. Using these equations, one can estimate the likely retention time of a variety of particles and understand what parameters control retention and separation in the system.

## 3 Materials and Methods

### 3.1 Instrumentation

All ElFFF experiments used a channel fabricated according to Shad [16], consisting of two graphite plates separated by a 25.4‑µm Mylar spacer with a 10 cm × 0.5 cm flow channel cut into it. CyElFFF was implemented using a single integrated setup composed of several instruments, including a high‐pressure liquid chromatography (HPLC) pump from Shimadzu (VP LC‐10AD) for carrier delivery, a CO_2_ suppressor (Metrohm 853) positioned downstream of the pump, a Rheodyne 9725i injector with a 2‐µL internal loop, an Agilent 33120A signal generator for applying the AC field, a Metrohm 945 Professional Detector Vario for conductivity measurement, and MagIC Net 3.2 software for data acquisition. The conductivity detector measured changes in total ionic conductivity and did not provide compound‐ or species‐specific identification. Conductivity features were therefore designated according to their elution order as the void response and the first and second delayed conductivity features. These designations do not establish the chemical identity, aggregation state, or number of PFAS populations represented by an individual feature.

### 3.2 Materials

Five ionic PFAS analytes were selected as representative compounds for this study: PFOS, PFHxS‐K, PFBS, GenX (hexafluoropropylene oxide dimer acid), which is a replacement for PFOA, and PFNA. These compounds were reagent‐grade and sourced from TCI, Sigma‐Aldrich, and JK Chemicals. These selected samples had concentrations ranging from 1 ppm to 20 ppm, which were achieved by diluting with Milli‐Q purified water. Notably, Milli‐Q purified water, characterized by a resistivity of 18.2 MΩ•cm at 25°C and a pH of 6.4–6.6, served as the carrier in all experimental procedures.

### 3.3 Experimental Conditions

Experiments were devised and carried out under controlled settings. All studies were conducted at temperatures ranging from 22 to 25°C. Unless specifically stated otherwise, a flow rate of 0.2 mL/min was used throughout all studies. The sample injection was 2 µL.

In this study, the retention behavior of several PFAS compounds (PFOS, PFBS, PFHxS‑K, GenX, and PFNA) was examined using CyElFFF under controlled flow and injection conditions. Unless otherwise noted, samples were 2 µL and analyzed using a 50% duty‑cycle square waveform. We first measured field‐off injections to establish the void peak (void time) (Figure 1), then measured retention profiles under an applied cyclical field (Figure 2). To evaluate field effects, we performed one‑factor sweeps while holding other conditions constant. For PFHxS‐K, voltage was applied at 2.0, 2.5, 3.0, and 3.5 Vpp at a fixed frequency of 2 Hz (Figure 3). The applied voltage was limited to ≤3.5 Vpp because increased baseline instability was observed when higher voltage settings were tested. Previous studies have shown that the electrochemical response of CyElFFF channels depends on the applied voltage and other operating conditions [13]. However, the cause of the instability observed in the present system was not independently determined. Electrode reactions, gas formation, local changes in pH or ionic composition, and instrumental effects are possible contributors, but these processes and the effective bulk‐channel electric field were not measured directly. Therefore, 3.5 Vpp is reported as an empirical operating limit for the present system rather than as a confirmed electrolysis threshold. For frequency experiments using PFHxS‑K, the frequency of the 3 Vpp waveform was set at 2, 5, and 10 Hz (Figure 4). PFHxS‑K was used as a representative mid‑chain sulfonate to study field‑dependent behavior.

**FIGURE 1:**
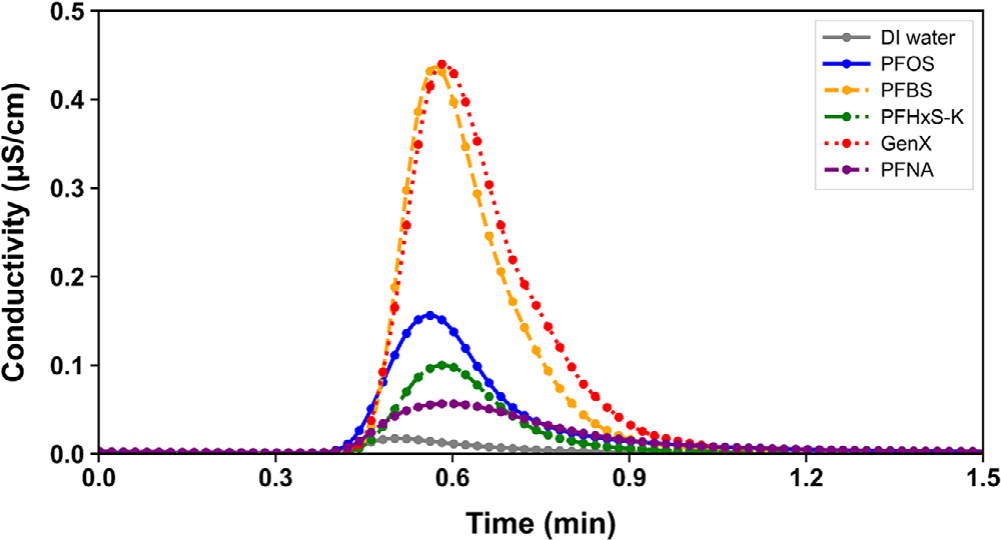
Void peaks of various per‐ and polyfluoroalkyl substances (PFAS) compounds obtained using Cyclical Electrical Field‐Flow Fractionation (CyElFFF) fractograms. The sample volume was 2 µL with a concentration of 2 ppm, with no voltage applied. All void peaks appeared after 0.4 min.

**FIGURE 2:**
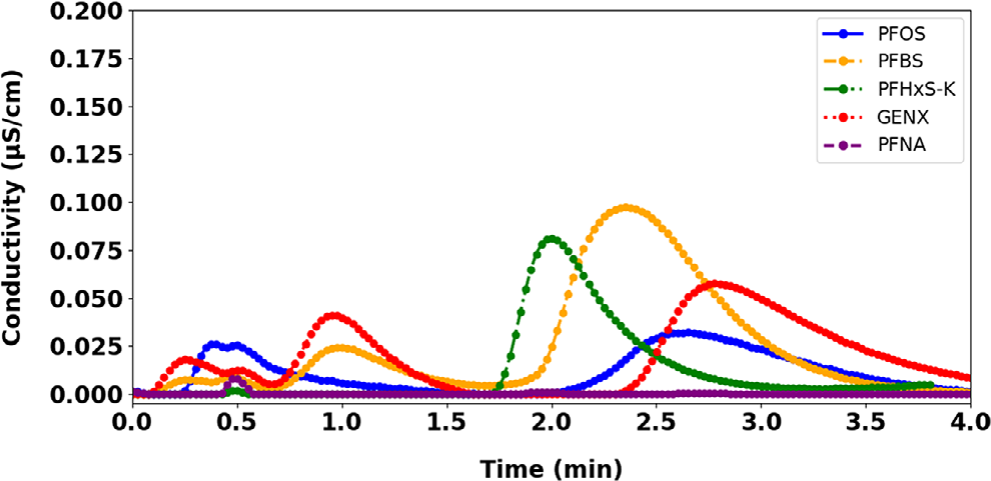
Retention profiles of various per‐ and polyfluoroalkyl substances (PFAS) types obtained using Cyclical Electrical Field‐Flow Fractionation (CyElFFF) fractograms. The sample volume was 2 µL with a concentration of 2 ppm. Experimental conditions included a 3 Vpp square wave, 2 Hz frequency, and a flow rate of 0.2 mL/min.

**FIGURE 3:**
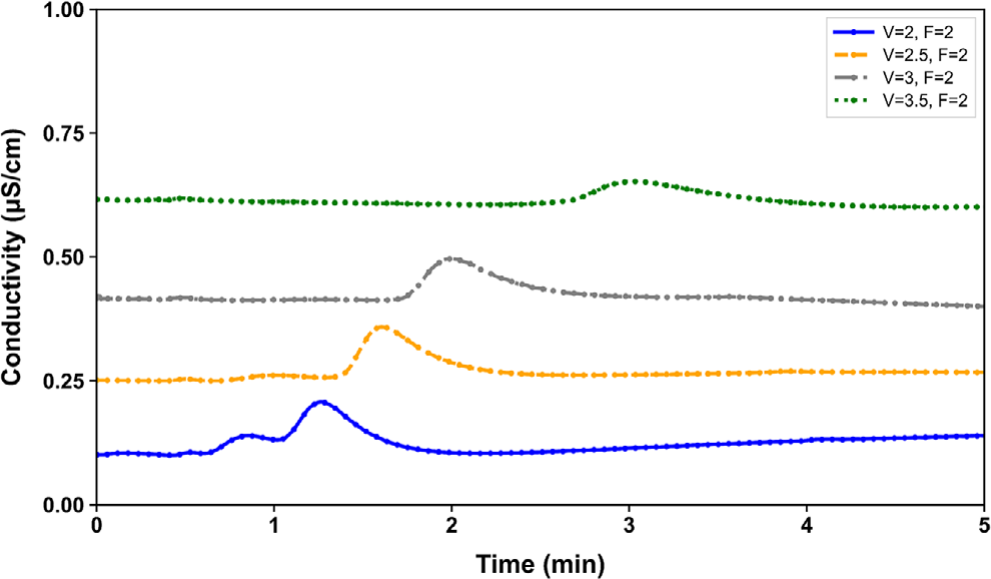
Cyclical Electrical Field‐Flow Fractionation (CyElFFF) fractograms illustrate the retention of perfluorohexane sulfonate (PFHxS‐K) at a concentration of 2 ppm for different electric fields. Conditions were 2, 2.5, 3, and 3.5 Vpp square wave, 2 Hz frequency, and a flow rate of 0.2 mL/min.

**FIGURE 4:**
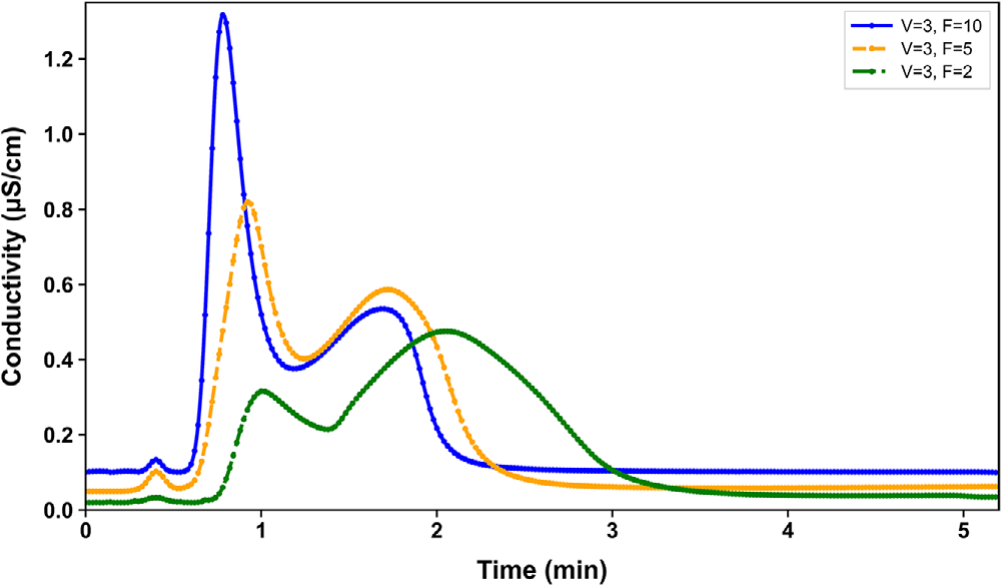
Cyclical Electrical Field‐Flow Fractionation (CyElFFF) fractograms illustrating the retention of perfluorohexane sulfonate (PFHxS‐K) at a concentration of 10 ppm for different electric fields. Conditions were 3Vpp square wave, 2, 5, and 10 Hz, and a flow rate of 0.2 mL/min.

To test mixture behavior, PFBS and PFHxS‑K were mixed at equal concentrations (2 ppm each) and analyzed at 3 Vpp and 2 Hz; individual injections under the same conditions were used as references for comparison (Figure 6). Finally, to examine concentration effects, PFBS was injected at 0.5, 1.0, and 2 ppm under fixed field conditions (3 Vpp, 2 Hz), and changes in peak height, apparent retention time, and peak broadening were compared (Figure 5). PFBS was selected as the probe analyte because its conductivity response was stable and reproducible under the tested conditions.

**FIGURE 5:**
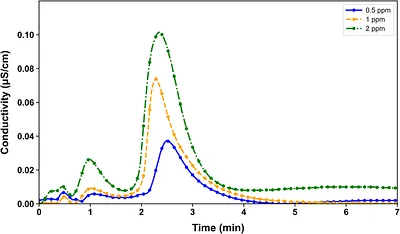
Cyclical Electrical Field‐Flow Fractionation (CyElFFF) fractograms illustrate the retention of perfluorobutanesulfonate (PFBS) at varying concentrations (0.5, 1, and 2 ppm) under a 3Vpp square wave and 2 Hz frequency. The flow rate was 0.2 mL/min.

### 3.4 Retention‐Ratio Calculation Method

For all retention‐ratio calculations, the observed retention time, *t_R,obs_*, was defined as the time at the peak apex. Corrected retention times were calculated as *t_R,corr_* = *t_R,obs_* ‐ *t_d_*, where the extra‐column transit‐time correction applied in this study was *t_d_* = 0.44 min. The detector‐observed deionized (DI)‐water void time was 0.5053 min, corresponding to a corrected void time of 0.0653 min. Each voltage, frequency, and concentration condition was evaluated using three traces (*n* = 3). Retention ratios were calculated independently for each trace as *R* = 0.0653/*t_R,corr_* and are reported as mean ± sample standard deviation (SD). When two retained peaks were resolved, each peak was evaluated separately.

### 3.5 Safety

The PFAS studied here (PFOS, PFHxS‐K, PFBS, GenX, and PFNA) are environmentally persistent, bioaccumulative, and toxic, and are regulated as emerging contaminants of concern. All PFAS standards, stock solutions, and PFAS‐containing carrier and waste were treated as hazardous material, segregated, and disposed of through the University of Utah's regulated hazardous waste program rather than to the laboratory drain. Routine precautions (nitrile gloves, eye protection, and solution preparation in a ventilated area) were used. No unexpected or unusually high hazards were encountered during this work.

### 3.6 Use of Artificial Intelligence‐assisted Tools

During preparation of this manuscript, the authors used Claude (Anthropic) to assist with language editing and figure formatting code. The authors reviewed and edited all Artificial Intelligence‐assisted content and take full responsibility for the accuracy and integrity of the published work.

## 4 Results and Discussion

### 4.1 Effect of Electric Field

The first set of experiments involved the observation of PFAS retention in the channel with and without an applied voltage. The results for PFOS, PFBS, PFHxS, GenX, and PFNA, with no voltage applied, are shown in Figure 1. Figure 1 shows that the maximum peak intensity for a blank injection of DI water is lower than all of the other samples, which is expected. Additionally, under field‐off conditions, all detected samples produced void peaks within approximately 0.50–0.60 min. The DI‐water reference void peak, which was used for the retention‐ratio correction, was centered at 0.505 min. The variation in signal intensity is due to the different materials injected, as their conductivity influences the magnitude of the maximum void peak, since the detector is a conductivity detector.

The results of the same samples but in the presence of an applied field are shown in Figure 2. In Figure 2, all sample injections showed a void peak at approximately 0.50–0.60 min. Occasional earlier signals were attributed to injection‐related baseline fluctuations. Under field‐on conditions, one or more delayed conductivity features appeared after the void peak, and their positions and relative prominence varied among the individual PFAS standard injections. For PFBS and GenX, the delayed features occurred closer to the void peak, whereas the PFNA response occurred later. For PFOS and PFHxS‐K, the earlier delayed feature was weak or not clearly resolved, while the later feature was dominant. These field‐dependent delayed responses are consistent with measurable retention under the applied CyElFFF conditions. However, although each experiment involved an individual PFAS standard, conductivity detection is non‐selective and cannot establish the specific chemical or physical origin of each delayed feature. The responses are therefore described operationally according to their elution order as earlier and later retained features.

The occurrence of more than one retained feature following injection of an individual PFAS standard represents a departure from the simplest ideal single‐mobility CyElFFF model. Under the ideal CyElFFF model, field‐driven transverse migration moves charged material toward slower‐flowing regions near the channel walls, thereby increasing residence time. In the present system, however, the graphite electrodes also serve as the channel walls. Field‐driven migration may therefore increase PFAS‐surface contact, allowing reversible adsorption/desorption or other wall‐mediated interactions to contribute to the observed retention. Because the electrophoretic mobilities of the PFAS analytes and the effective electric field within the channel were not independently determined, and because the present experiments do not isolate surface interactions, the relative contributions of ideal field‐driven migration and wall‐mediated retention cannot be quantified. Mobility heterogeneity, aggregation or association, and reversible surface interactions remain possible contributors to the observed multiple retained features, but these mechanisms cannot be distinguished from the present data and are therefore considered hypotheses requiring independent verification. Accordingly, the individual retained features are not assigned to aggregates, distinct mobility populations, or separate ideal Mode III trajectories.

In all experiments, after 5 min, the field was switched off to probe the field‐off response. A release peak appeared immediately after field‐off for all analytes, suggesting some materials were still retained in the channel. Table 1 summarizes the integrated conductivity‐detector response obtained during the field‐on portion of each experiment and the integrated response observed after removal of the field. Both values were normalized to the integrated response from the corresponding field‐off control. Because conductivity detection is non‐selective and no compound‐specific PFAS calibration or independent mass quantification was performed, these percentages represent relative integrated detector responses rather than PFAS mass recoveries.

**TABLE 1:** Relative integrated conductivity‐detector responses normalized to the corresponding field‐off controls.

Analyte	Field‐on integrated conductivity‐detector response relative to the field‐off control (%)	Post‐field‐off integrated conductivity‐detector response relative to the field‐off control (%)
PFOS	95.15%	0.46%
PFBS	76.02%	0.22%
PFHxS‐K	91.60%	0.38%
Gen X	77.28%	0.38%
PFNA	97.36%	0.68%

The post‐field‐off feature represented only a small fraction of the conductivity response measured for the corresponding field‐off control. This observation indicates that a small detector response occurred after removal of the field; however, it does not establish the chemical identity of the responding material or quantify the amount of PFAS retained within or released from the channel. Because these post‐field‐off features were small relative to the principal responses, this region is not shown in the main fractograms for clarity. The corresponding relative detector‐response values are reported in Table 1.

### 4.2 The Impact of Varying Voltage While Maintaining a Constant Frequency

The effect of increasing voltage on PFHxS‐K retention was examined while maintaining a constant frequency and is shown in Figure 3. For visual clarity, the conductivity traces were vertically offset. No horizontal shifting, time‐axis normalization, or modification of the retention‐time positions was applied. The vertical offsets were used only for graphical presentation and therefore did not affect the retention‐time or retention‐ratio calculations. Under these conditions, the traces show an early void response followed by one or two retained features depending on the applied voltage. The presence of a second retention peak in some runs is unexpected based on the single‐mobility behavior discussed previously. At 2.0 and 2.5 Vpp, two distinct retention peaks are observed, suggesting the presence of two effective migration states, possibly arising from weak wall interactions or mobility heterogeneity. However, when the voltage is increased to 3.0 and 3.5 Vpp, the earlier retention peak disappears and only a single dominant retention band remains. The relative prominence of the earlier and later retained features changed with increasing applied voltage. Because the conductivity detector does not identify the material responsible for each feature, the reduction or disappearance of the earlier feature cannot be assigned uniquely to the collapse of multiple mobility populations. Possible contributions include changes in transverse migration, wall interactions, feature overlap, or the relative magnitude of unresolved conductivity responses. Quantitatively, the mean retention ratio decreases systematically with increasing voltage as shown in Table 2. For the first retention peak, the mean retention ratio decreases from approximately 0.157 at 2.0 Vpp to 0.117 at 2.5 Vpp. Similarly, for the second retention peak, the mean retention ratio decreases from 0.079 to 0.024 as the voltage increases from 2.0 to 3.5 Vpp. The decreasing retention ratio indicates greater net retention with increasing applied voltage. The decrease in mean retention ratio with increasing applied voltage is qualitatively consistent with the trend predicted for Mode III CyElFFF. In this mode, a stronger electric field reduces the time particles spend in the middle of the channel and pushes particles closer to the channel walls, where the flow velocity is slower. This causes the particles to take longer to traverse the length of the channel, leading to an extended retention time, as expected. However, increasing voltage may also increase wall contact or alter interfacial retention, so the trend does not independently establish ideal Mode III transport.

**TABLE 2:** Retention ratios (*R*) for PFHxS‐K at different applied voltages. Retention ratios were calculated using the procedure described in the Methods section. NR indicates that the corresponding retained feature was not resolved.

Electrical condition	*R*, first retained peak (mean ± SD, *n* = 3)	*R*, later retained peak (mean ± SD, *n* = 3)
*V* = 2.0 Vpp, *f* = 2 Hz	0.15699 ± 0.00048	0.07912 ± 0.00008
*V* = 2.5 Vpp, *f* = 2 Hz	0.11703 ± 0.00776	0.05692 ± 0.00274
*V* = 3.0 Vpp, *f* = 2 Hz	NR	0.04183 ± 0.00251
*V* = 3.5 Vpp, *f* = 2 Hz	NR	0.02478 ± 0.00078

### 4.3 The Impact of Varying Frequency While Maintaining a Constant Voltage

The effect of frequency on PFHxS‐K retention can be observed in Figure 4. At 2, 5, and 10 Hz, PFHxS‐K shows clear retention, eluting as a distinct retained peak. Decreasing the frequency from 10 Hz to 2 Hz resulted in a small increase in retention time (Figure 4). As observed in all samples, the void peaks appeared at around 0.5 min, followed by earlier and later delayed conductivity features. This behavior is qualitatively consistent with Mode III dynamics, in which increasing the frequency reduces the time available for cross‐channel migration, resulting in shorter retention times. However, because the analyte electrophoretic mobility and effective bulk‐channel field were not determined, $\;\lambda_{0}$ could not be calculated directly; therefore, these observations do not confirm Mode III operation. Also, as the frequency increased, the height of the retention peaks increased. This behavior can be explained by the influence of frequency on band broadening in CyElFFF. Previous studies have shown that frequency can affect peak shape and broadening, with diffusion playing an important role in this process [11].

Quantitatively, the retention ratio shows a noticeable increase for the first retention peak with increasing frequency at constant voltage (3 Vpp), as shown in Table 3. Specifically, the mean retention ratio increases from approximately 0.116 at 2 Hz to 0.133 at 5 Hz and further to 0.188 at 10 Hz. In contrast, the second retention peak exhibits only a slight variation, with the mean retention ratio increasing marginally from approximately 0.040 at 2 Hz to 0.050 at 5 Hz and 0.052 at 10 Hz. This relatively small change in the mean retention ratio suggests that, under a fixed electric field strength (3 Vpp), increasing frequency has a weaker influence on retention when compared to voltage variation.

**TABLE 3:** Retention ratios (*R*) for PFHxS‐K at different applied frequencies. Retention ratios were calculated using the procedure described in the Methods section.

Electrical conditions	*R*, first retained peak (mean ± SD, *n* = 3)	*R*, later retained peak (mean ± SD, *n* = 3)
*V* = 3, *f* = 2	0.11625 ± 0.02060	0.04050 ± 0.00254
*V* = 3, *f* = 5	0.13346 ± 0.01062	0.05084 ± 0.00156
*V* = 3, *f* = 10	0.18839 ± 0.01788	0.05202 ± 0.00139

### 4.4 Effect of Concentration on Retention Time

To investigate the effect of concentration on retention time, a series of experiments was conducted using PFBS as the analyte. The experimental conditions were held constant at 3Vpp and 2 Hz, while the concentration of PFBS was varied systematically (Figure 5). The data indicate that as the concentration increases from 0.5 ppm to 2 ppm, the height of the retention peaks grows, indicating an increased number of particles being detected, as expected. Across all concentrations, a void peak appears at ∼0.5 min, followed by a first retention peak at ∼1.0 min. The second retained peak appears within a similar time window for all concentrations (approximately 2.3–2.5 min), with only small shifts in peak position. Notably, the 0.5 ppm trace shows slightly longer retention compared with the higher‐concentration traces. This behavior is consistent with the expectation that higher sample loading can mildly reduce retention due to overloading effects, in which the analyte is displaced into faster flow streamlines and therefore elutes sooner.

Overall, concentration primarily affects signal magnitude. As PFBS concentration increases, the retention peak height increases, consistent with a larger injected analyte mass. In contrast, retention ratios and peak positions change only slightly over the tested concentration range, indicating that retention behavior remains relatively stable under these conditions.

Quantitatively, the retention ratio shows only minor variation with increasing concentration, as summarized in Table 4. For the first retained peak, the mean retention ratio increased from approximately 0.068 at 0.5 ppm to 0.101 at 1 ppm and 0.109 at 2 ppm. For the second retained peak, the mean retention ratio values were approximately 0.030, 0.033, and 0.033 at 0.5, 1, and 2 ppm, respectively, indicating relatively little change in the retention ratio of the second peak over the investigated concentration range.

**TABLE 4:** Retention ratios (R) for perfluorobutanesulfonate (PFBS) at different concentrations.

Electrical conditions	*R*, first retained peak (mean ± SD, *n* = 3)	*R*, second retained peak (mean ± SD, *n* = 3)
[*C*] = 0.5 ppm	0.06842 ± 0.00974	0.03041 ± 0.00069
[*C*] = 1ppm	0.10192 ± 0.01279	0.03369 ± 0.00185
[*C*] = 2 ppm	0.10951 ± 0.01360	0.03324 ± 0.00134

Overall, these small changes indicate that concentration does not significantly influence retention behavior. Instead, concentration mainly affects peak height, while peak position and retention ratio remain largely unchanged. These results highlight the importance of controlling sample concentration to maintain consistent signal intensity and reproducible measurements.

### 4.5 Mixed PFAS Experiment

PFBS and PFHxS‐K were tested separately and as a binary mixture at 3 Vpp and 2 Hz. Each individual standard contained 2 ppm of the corresponding PFAS, whereas the mixture contained 2 ppm PFBS and 2 ppm PFHxS‐K, giving a total PFAS concentration of 4 ppm. As shown in Figure 6, the individual PFBS injection produced a broad conductivity response with a dominant delayed feature near 2.3 min, while the PFHxS‐K response appeared near 2.4 min. The overlap between these responses suggested that the tested conditions offered limited selectivity between the two compounds.

**FIGURE 6:**
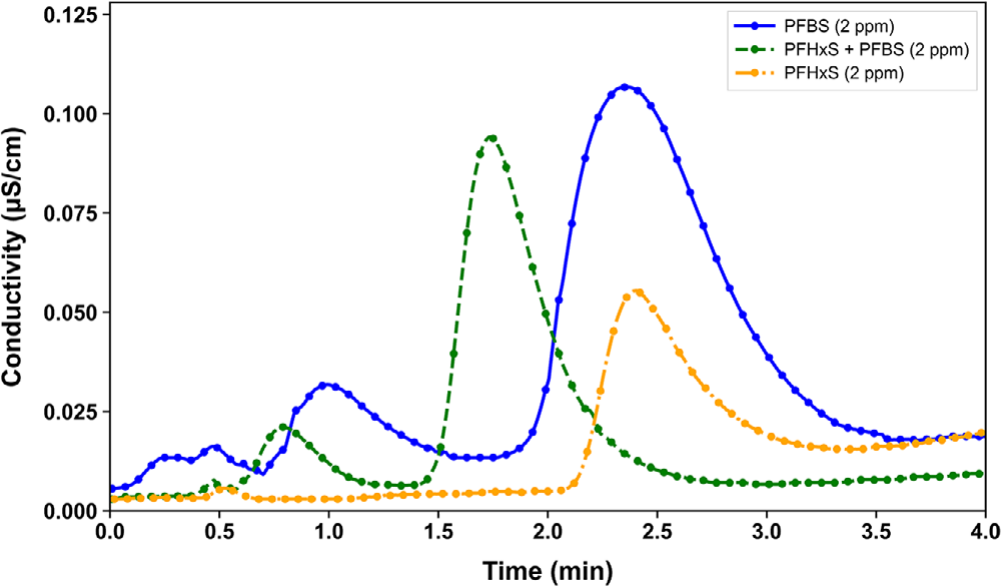
Cyclical Electrical Field‐Flow Fractionation (CyElFFF) fractograms of perfluorobutanesulfonate (PFBS) (2 ppm), perfluorohexane sulfonate (PFHxS‐K) (2 ppm), and a 1:1 mixture under a 3 Vpp square wave at 2 Hz with 0.2 mL min^−^^1^ deionized (DI) carrier.

The mixed injection produced earlier, partially merged conductivity signals and did not demonstrate baseline separation. However, the individual standards and the mixture were not matched for total PFAS concentration, conductivity, or ionic strength [17]. Therefore, the differences in peak position and shape cannot be attributed specifically to interactions between PFBS and PFHxS‐K. In addition, the non‐selective conductivity detector cannot determine which compound contributed to each signal in the mixed profile. This experiment is therefore presented as a preliminary observation. Concentration‐ and conductivity‐matched controls, together with compound‐selective analysis, would be required to determine the cause of the altered mixture profile and evaluate the separation more reliably.

## 5 Conclusion

This study explored the retention behavior of PFOS, PFHxS‐K, PFBS, GenX, and PFNA using CyElFFF. Field‐off experiments established the void response, while application of a cyclical electric field produced reproducible delayed conductivity responses following PFAS standard injections, demonstrating that the applied field influenced analyte transport through the channel under the conditions tested.

Applied voltage had a clear effect on retention, with higher voltages generally producing longer retention times and lower retention ratios. Increasing frequency produced smaller but measurable changes, generally resulting in shorter retention times and higher retention ratios. These voltage‐ and frequency‐dependent trends are qualitatively consistent with the expected retention behavior of CyElFFF, including Mode III behavior. However, the operating mode was not quantitatively established because the electrophoretic mobilities of the analytes and the effective bulk electric field within the channel were not independently determined; therefore, the dimensionless operating parameter, λ_0_, could not be calculated.

The occurrence of multiple retained features for nominally single PFAS standards also represents a departure from the simplest ideal single‐mobility CyElFFF model. Field‐driven migration toward slower‐flowing regions near the channel walls may contribute to retention, but increased contact with the graphite channel surfaces may also introduce reversible adsorption/desorption or other wall‐mediated interactions. The relative contributions of these mechanisms cannot be determined from the present measurements. Because conductivity detection is non‐selective, the individual retained features cannot be assigned to specific PFAS forms, aggregates, or distinct mobility populations. Increasing PFBS concentration primarily increased the conductivity signal, while retention ratios and peak positions changed only slightly over the tested range. PFBS and PFHxS‐K were not baseline resolved under the investigated CyElFFF conditions (3 Vpp, 2 Hz). Although the binary mixture produced shifted and partially overlapping conductivity signals, differences in total PFAS concentration, conductivity, and ionic strength between the individual and mixed samples prevent these changes from being attributed to specific PFAS–PFAS interactions. Therefore, the mixture experiment should be considered exploratory, and no mechanistic conclusions can be drawn from these data.

Despite these limitations, this study demonstrates the potential of CyElFFF for investigating the field‐dependent behavior of PFAS and identifies several factors that require further investigation. Future work should include concentration‐ and conductivity‐matched controls, calibration of the apparent electric field within the CyElFFF channel, independent measurements of electrophoretic mobility under the experimental carrier conditions, systematic evaluation of channel‐surface interactions, compound‐selective analysis (e.g., LC‐MS), determination of analytical detection limits, and further optimization of the carrier composition, applied voltage, frequency, and waveform. Addressing these factors will provide a more reliable understanding of PFAS transport and retention in CyElFFF and improve the interpretation of future separation studies.

## Author Contributions

**Fatemeh Momeni**: conceptualization, investigation, formal analysis, visualization, and writing (original draft). **Jenika Mifflin**: investigation and writing (review and editing). **Himanshu Sant**: methodology, supervision, and writing (review and editing). **Bruce K. Gale**: conceptualization, methodology, supervision, funding acquisition, and writing (review and editing).

## Funding

Partial funding for this project was provided by the “Catalyst Grant”, NSF Futures Engine in the Southwest to the Sant Laboratories at the University of Utah.

## Conflicts of Interest

The authors declare no conflicts of interest.

## Data Availability

The data that support the findings of this study are available from the corresponding author upon reasonable request.
